# Definition and Characterization of SOX11-Derived T Cell Epitopes towards Immunotherapy of Glioma

**DOI:** 10.3390/ijms24031943

**Published:** 2023-01-18

**Authors:** Yibin Liu, Anna Keib, Brigitte Neuber, Lei Wang, Angelika B. Riemer, Maria Bonsack, Angela Hückelhoven-Krauss, Anita Schmitt, Carsten Müller-Tidow, Michael Schmitt

**Affiliations:** 1 Department of Internal Medicine V, Heidelberg University Hospital, Im Neuenheimer Feld 410, 69120 Heidelberg, Germany; 2 Immunotherapy and Immunoprevention, German Cancer Research Center (DKFZ), 69120 Heidelberg, Germany; 3 Molecular Vaccine Design, German Center for Infection Research (DZIF), Partner Site Heidelberg, 69120 Heidelberg, Germany

**Keywords:** SOX11, glioma, HLA-A*0201, tumor-associated antigen, immunotherapy

## Abstract

The transcription factor SOX11 is a tumor-associated antigen with low expression in normal cells, but overexpression in glioblastoma (GBM). So far, conventional surgery, chemotherapy, and radiotherapy have not substantially improved the dismal prognosis of relapsed/refractory GBM patients. Immunotherapy is considered a promising strategy against GBM, but there is a fervent need for better immunotargets in GBM. To this end, we performed an in silico prediction study on SOX11, which primarily yielded ten promising HLA-A*0201-restricted peptides derived from SOX11. We defined a novel peptide FMACSPVAL, which had the highest score according to in silico prediction (6.02 nM by NetMHC-4.0) and showed an exquisite binding affinity to the HLA-A*0201 molecule in the peptide-binding assays. In the IFN-γ ELISPOT assays, FMACSPVAL demonstrated a high efficiency for generating SOX11-specific CD8^+^ T cells. Nine out of thirty-two healthy donors showed a positive response to SOX11, as assessed by the ELISPOT assays. Therefore, this novel antigen peptide epitope seems to be promising as a target for T cell-based immunotherapy in GBM. The adoptive transfer of in vitro elicited SOX11-specific CD8^+^ T cells constitutes a potential approach for the treatment of GBM patients.

## 1. Introduction

Brain tumors can arise in all age groups, being especially devastating in children, and they are the leading cause of cancer-related mortality and morbidity [1]. Glioma is one of the most common primary tumors in the brain, accounting for about 81% of central nervous system (CNS) malignancies [2]. The most common subtype of glioma is GBM, which has a 5-year survival rate of ~5% [3]. The blood–brain barrier prevents the majority of antitumor drugs from entering the brain. This constitutes a major obstacle for the development of antiglioma drugs [4]. Conventional surgery, chemotherapy, and radiotherapy have made limited improvements in the prognosis of glioma patients [5]. Because of its minimal side effects, tumor-specific cytotoxicity, and durable antitumor effect, T cell-based immunotherapy is regarded as a promising therapeutic approach for incurable malignant gliomas [6]. However, endogenously activated T cells induced by immunotherapies such as vaccination may not be sufficient to control tumor cells, since the tumor-specific antigens can serve as self-antigens or the tumor can escape the immune response through its immune-evasion mechanisms [7]. Recently, a large number of immunotherapeutic clinical trials with tumor cell or dendritic cell (DC) vaccines for glioma patients have been performed [6,8]. However, these trials failed to effectively expand tumor-antigen-specific T cells, suggesting that the endogenous activation of T cells is insufficient to control tumors [6,8]. A strategy to overcome these limitations is adoptive T cell therapy, in which tumor-specific T cells are rapidly expanded ex vivo and then transferred to patients. In this way, peripheral blood mononuclear cells (PBMCs) are repetitively stimulated by tumor-associated antigens presented by antigen-presenting cells (APCs) such as DCs [9]. These cells can be expanded rapidly ex vivo for use in adoptive T cell therapy [10]. Some glioma-associated antigens have been identified over the past few decades, but the antigens most suitable for expanding tumor-specific T cell responses are still under investigation [6]. Cytotoxic T cells recognize tumor cells via the binding of T cell receptors (TCRs) to peptides presented on the cell surface by major histocompatibility complex (MHC) class-I molecules. Peptide binding to MHC molecules is the most selective step in the antigen presentation pathway. The MHC is also known as the human leukocyte antigen (HLA) in humans. There are thousands of different HLA class-I molecules, every human being carries three to six variants, and every HLA molecule binds different peptides. Peptides bind to MHC molecules via anchor residues, and binding motifs can be defined for the various peptides binding to a given MHC molecule. These can be used to predict peptide–MHC binding. One of the first predictors developed was SYFPEITHI [11], based on a collection of MHC class-I and class-II ligands and peptide motifs; more recent developments are the NetMHC family of predictors, based on both binding affinity and eluted MHC ligand data [12,13,14,15,16,17].

SOX genes are developmental regulators that play an important role in neurogenesis, neural crest development, hematopoiesis, chondrogenesis, and stem-cell maintenance. They are also significant for tissue homeostasis and regeneration in adults, particularly in the CNS [18]. The accumulation of genetic mutations in different cell types of the CNS may induce gliomas [19]. The dysfunction and mutation of SOX factors are implicated in a variety of malignancies [9]. There is evidence that the expression of SOX11 is reactivated during tumorigenesis [20]. Moreover, Schmitz et al. identified an immunogenic CD8^+^ T cell epitope derived from SOX11 [21], which emphasized the suitability of SOX11 as a promising novel target for the immunotherapy of malignant gliomas. Although antigen-targeted immunotherapy has enormous potential to elicit antigen-specific antitumor effects, the lack of effective targets remains a significant obstacle in effectively and safely treating glioblastoma and other malignant gliomas with low mutation loads [22].

The aim of this study was to investigate the suitability of SOX11 peptides as a target antigen for the immunotherapy of glioma. We predicted and experimentally verified HLA-A*A0201 binders and analyzed the IFN-γ secretion of T cells after priming by peptide-pulsed mature DCs in healthy donors using ELISPOT assays and flow cytometry. An immunogenic HLA-A*0201-restricted peptide derived from SOX11 was proved to be effective in activating tumor-specific CD8^+^ T cells response. This novel CD8^+^ T cell epitope may serve as an attractive candidate for a T cell-based immunotherapy for glioma.

## 2. Results

The immunotherapy of GBM relies on well-defined immunogen peptides. In order to identify such peptides, we performed an in silico prediction and selected ten promising HLA-A*0201-restricted peptides derived from SOX11. The novel peptide FMACSPVAL, which had the highest prediction score, showed a strong binding affinity to the HLA-A*0201 molecule in vitro. We compared two different methods for generating peptide-specific CD8^+^ T cells. In the IFN-γ ELISPOT assays, FMACSPVAL demonstrated a high efficiency for generating SOX11-specific CD8^+^ T cells. After stimulation, the FMACSPVAL-specific CD8^+^ T cells showed an increase in TNF-α secretion, as detected by intracellular cytokine staining. Additionally, the killing of a target cell line expressing SOX11 was observed.

### 2.1. Peptides Derived from SOX11 as Defined by In Silico Prediction

In order to determine whether SOX11 could serve as a molecular target antigen for glioma-specific CD8^+^ T cells, potential HLA-A*0201 ligands were selected from the amino acid sequence of SOX11 using 11 HLA ligand prediction algorithms, identified through the MHC combine tool (https://mhccombine.dkfz.de/mhccombine/, accessed on 14 July 2017, and again on 2 May 2022) [23]. We further included the peptide LLRRYNVAKV from SOX11, which was previously described as a potential HLA-A*0201 ligand [21], even though it was only predicted to be a weak or non-HLA-A*0201 binder in our study. The MART-1 analogue peptide (ELAGIGILTV) was used as a positive control [24].

We chose the nine most promising HLA-A*0201-binding peptides from the various predictions, sorted according to the NetMHC-4.0 binding affinity results in Table 1. Among the nine peptides, FMACSPVAL had a NetMHC-4.0 predicted binding affinity of 6.02 nM and was therefore regarded as the most promising peptide. All eleven peptides were synthesized and used for in vitro HLA-A*0201-binding assays and the generation of peptide-specific CD8^+^ T cells.

### 2.2. Experimental HLA-A*02-Binding Affinity of Predicted Peptides on T2 Cells

**Figure 1:**
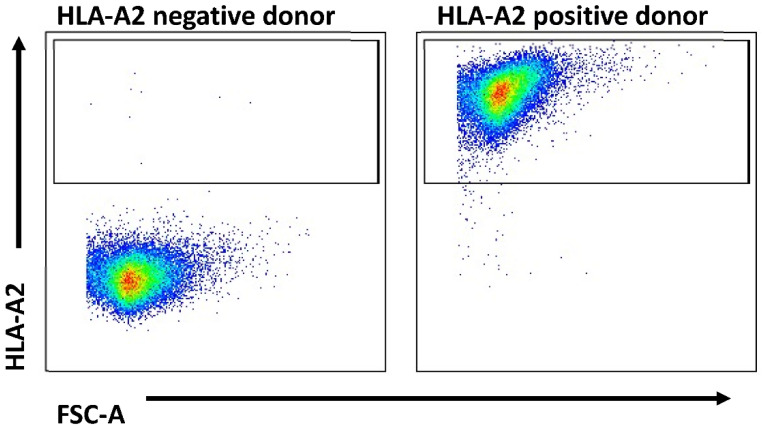
HLA typing of PBMC samples. We predicted SOX11 peptides that bind with HLA-A*0201; therefore, healthy donors were tested for the HLA-A*0201 molecule. The figure shows dot blot results obtained by flow cytometry for an exemplary HLA-A*0201 staining assay. All PBMCs were tested.

To evaluate the binding affinity of the ten SOX11 peptides (one previously published peptide (LLRRYNVAKV) and nine peptides identified by in silico prediction, see Table 1) to HLA-A*0201 molecules, a peptide-binding assay was performed on T2 cells in vitro, with detection via flow cytometry. For all experiments, samples from healthy donors were used, and HLA typing was performed (Figure 1). The peptide ELAGIGILTV was used as a positive control, and cells without peptides were used as a negative control. 

**Figure 2:**
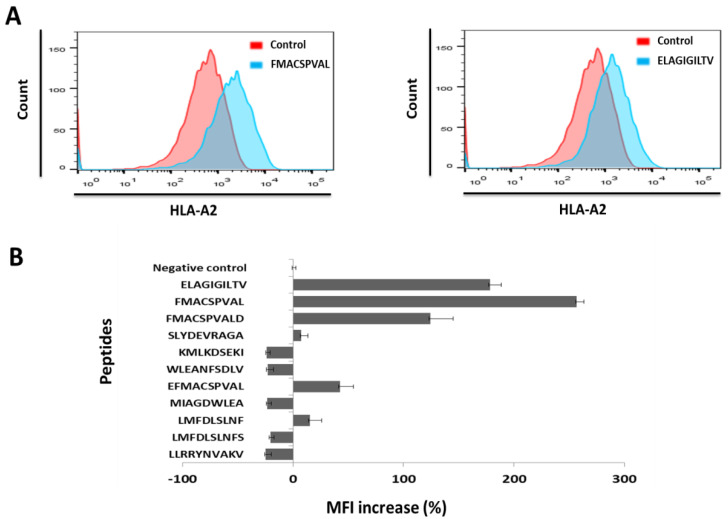
HLA-A*0201-binding affinity assay for different peptides on T2 cells (18 h). To evaluate the binding affinity of each candidate peptide to the HLA-A*0201 molecules, a T2 peptide-binding assay was performed. The upregulation of HLA-A*0201 molecules on T2 cells may be detected by fluorescence intensity exchange, which reflects the peptide-binding ability to HLA-A*0201 molecules. The peptide ELAGIGILTV was used as a positive control, and cells without peptides were used as a negative control. (**A**) Flow cytometry results of peptides FMACSPVAL and ELAGIGILTV shown in red and the negative control shown in blue. (**B**) The binding activity of all 11 peptides to HLA-A*0201 molecules was determined semi-quantitatively by measuring the peptide-induced expression of HLA-A*0201 on T2 cells with flow cytometry. Unloaded T2 cells were considered as a negative control. Mean values were calculated for each group; error bars indicate SD.

As shown in Figure 2, the binding affinity of the peptide FMACSPVAL was much higher than that of the peptide ELAGIGILTV and the peptide LLRRYNVAKV (previously reported in [21]). The % MFI increase of the peptide FMACSPVAL was 256.8%, while the % MFI increase of the peptides ELAGIGILTV and LLRRYNVAKV were 178.2% and −24.6%, respectively. The peptides FMACSPVALD, EFMACSPVAL, SLYDEVRAGA, and LMFDLSLNF had % MFI increases of 124.2%, 42.5%, 7.4%, and 15.1%, respectively. Other predicted peptides did not have a higher binding affinity than the negative control. Of the nine newly predicted binders, the most strongly predicted peptide FMACSPVAL indeed proved to be a strong binder, even stronger than the positive control. The peptide FMACSPVALD also showed robust binding. Two peptides (EFMACSPVAL, LMFDLSLNF) showed intermediate binding, and one (SLYDEVRAGA) showed binding just above the background value. It is interesting to note that EFMACSPVAL was predicted to be a strong binder by NetMHC-4.0, but only predicted to be a binder at all by one more prediction algorithm, indicating that something may be questionable with this specific NetMHC-4.0 prediction. All other peptides were non-binders in the experimental assay, including the previously published peptide LLRRYNVAKV.

The peptide FMACSPVAL, which had the highest prediction scores out of the nine newly predicted binders, proved to be a stronger binder than the positive control. The peptide FMACSPVALD also showed robust binding. Two peptides (EFMACSPVAL and LMFDLSLNF) showed intermediate binding, and one (SLYDEVRAGA) showed binding just above the background value. We would like to point out that the peptide EFMACSPVAL was predicted to be a strong binder according to NetMHC-4.0 but not the other prediction algorithm. This result indicates that a careful comparison of the prediction tools’ analyses and an experimental confirmation are essential. All other peptides were non-binders in the experimental assay, including the previously reported peptide LLRRYNVAKV.

### 2.3. In Vitro Generation of CD8^+^ T Cells Recognizing the SOX11-Derived Peptides

As the predictions and experimental binding assays did not yield concordant results in all cases, we decided to include all predicted peptides in the T cell assays. In this study, we used two methods to generate the peptide-specific CD8^+^ T cells. We call our first method for generating peptide-specific CD8^+^ T cells the long method. In the long method, T cells were isolated from different healthy-donor blood samples and were weekly stimulated with peptide-pulsed mature DCs. After two stimulation cycles, peptide-specific CD8^+^ T cells were evaluated via IFN-γ ELISPOT assays. In the second method, called the short method, cryopreserved PBMCs from different healthy-donor blood samples were thawed and cultured with selected peptides and cytokines. This allowed for the generation of DCs within the PBMCs without prior separation. The cells were tested for the presence of peptide-specific CD8^+^ T cells by IFN-γ ELISPOT assays and intracellular flow cytometry staining. We did not perform a re-stimulation in this setting. All eleven peptides, including one positive peptide (ELAGIGILTV), one previously reported peptide (LLRRYNVAKV), and nine peptides identified by in silico prediction, were used for the in vitro generation of peptide-specific CD8^+^ T cells. As shown in Table 2, for the positive-control peptide ELAGIGILTV, three of the four healthy-donor samples presented positive antigen-specific responses, as detected by the IFN-γ ELISPOT assay, after stimulation via the long method, and eight out of eighteen healthy donors presented positive antigen-specific responses after stimulation via the short method. Therefore, the long method was more likely to generate peptide-specific CD8^+^ T cells than the short method.

For the peptides FMACSPVAL and LLRRYNVAKV, the ratios of the number of healthy donors showing positive responses to all healthy donors were 6:14 and 1:14, respectively, according to the ELISPOT assay when using the long method, whereas the ratios were 3:18 and 1:8, respectively, when using the short method. FMACSPVAL-specific CD8^+^ T cells demonstrated the highest immunogenic capability of all tested peptides, including the previously described peptide LLRRYNVAKV, no matter the method used (Table 2). In addition, FMACSPVAL-specific CD8^+^ T cells were also evaluated by intracellular flow cytometry staining. One out of ten healthy donors displayed higher TNF-α and IFN-γ production after stimulation with the peptide FMACSPVAL compared to the negative control. Taken together, the peptide FMACSPVAL had the highest score according to in silico prediction, the highest binding affinity to the HLA-A*0201 molecules according to the peptide-binding assay on T2 cells, and the highest efficiency for generating SOX11-specific CD8^+^ T cells according to the IFN-γ ELISPOT assay among the ten selected peptides, including the previously reported peptide LLRRYNVAKV.

### 2.4. Recognition of Peptide LLRRYNVAKV by T Cells from a Healthy Donor

**Figure 3:**
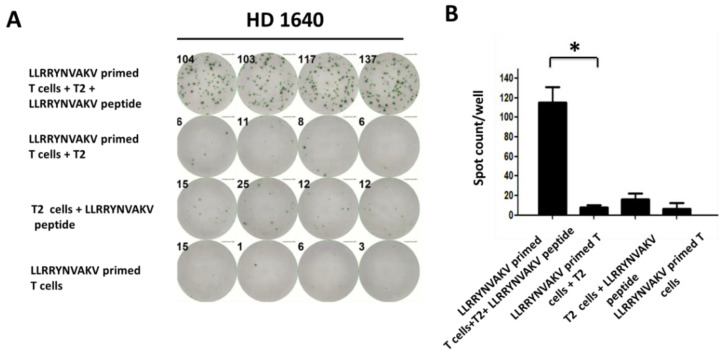
Recognition of peptide LLRRYNVAKV by T cells from a healthy donor (HD 1640). T cells from healthy donors were weakly stimulated with SOX11-derived LLRRYNVAKV-pulsed mature DCs. After two stimulation cycles, T cell cultures were detected for the activation of peptide-specific T cells. A total of 2 × 10^4^ primed T cells were added to 1 × 10^5^ peptide-loaded T2 target cells/well at an effector-cell-to-targeT cell ratio of 1:5. Primed T cells added to unloaded T2 cells served as a negative control group. Primed T cells and peptide-loaded T2 cells served as a background group. (**A**) IFN-γ ELISPOT data showing activated LLRRYNVAKV-specific T cells from one healthy donor. (**B**) The results are presented as the mean values of quadruplicate determinations. Mean values were calculated for each group; error bars indicate SD. Statistical significance was calculated using a paired-sample *t*-test and is indicated by * for *p* values < 0.05.

In a previous study, the SOX11-derived peptide LLRRYNVAKV was considered as a potential HLA-A*0201 ligand to induce glioma-specific CD8^+^ T cells [21]. Schmitz et al. tested T cell cultures for the presence of peptide-specific CD8^+^ T cells by chromium release assays after stimulation with the peptide LLRRYNVAKV. In our study, T cells from different healthy donors were weakly stimulated with LLRRYNVAKV-pulsed mature DCs. After two stimulation cycles, T cell cultures were detected for the activation of peptide-specific T cells by an IFN-γ ELISPOT assay. Primed CD8^+^ T cells were added to peptide-loaded T2 target cells at an effector-to-target ratio of 1:5. Primed T cells added to unloaded T2 cells served as a negative control group. Primed T cells and peptide-loaded T2 cells served as a background group. We observed a significant peptide-specific CD8^+^ T cell response stimulated by peptide-loaded T2 cells, as indicated by IFN-γ secretion in the ELISPOT assay (Figure 3A). The results are presented as the mean values of quadruplicate determinations (Figure 3B, *p* < 0.001). 

### 2.5. Recognition of Peptides FMACSPVAL, LMFDLSLNF, and SLYDEVRAGA by T Cells from Healthy Donors

**Figure 4:**
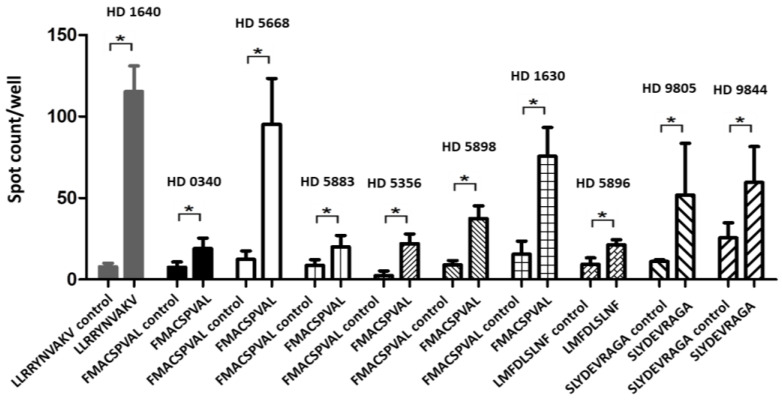
Summary of the positive peptide-specific T cell responses detected by IFN-γ ELISPOT for each healthy donor (HD). This figure shows only the positive responses of LLRRYNVAKV, FMACSPVAL, LMFDLSLNF, and SLYDEVRAGA peptide-specific T cells after a co-culture with T2 cells loaded with the respective peptides. Peptide-specific T cells co-cultured with unloaded T2 cells were considered as a negative control. The spots were counted, and the results were considered positive when the spot count was at least twice as high as that of the negative control. Mean values were calculated for each group; error bars indicate SD. Statistical significance was calculated using a paired-sample *t*-test and is indicated by * for *p* values < 0.05.

As mentioned above, the peptide FMACSPVAL had the highest score according to the in silico prediction, the highest binding affinity to HLA-A*0201 molecules according to the peptide-binding assay on T2 cells, and the highest efficiency for generating SOX11-specific CD8^+^ T cells according to the IFN-γ ELISPOT assay among the ten selected peptides, including the previously reported peptide LLRRYNVAKV [21]. Figure 4 shows the significant peptide-specific CD8^+^ T cell responses in the IFN-γ ELISPOT assay, with 48 h stimulation from FMACSPVAL-loaded T2 cells for six different healthy donors. Primed T cells added to unloaded T2 cells served as a negative control group. 

In addition, the IFN-γ ELISPOT data also showed SLYDEVRAGA-specific CD8^+^ T cell responses from two healthy donors and an LMFDLSLNF-specific CD8^+^ T cell response from one healthy donor (Figure 4). In this study, we found that three new peptides (FMACSPVAL, LMFDLSLNF, and SLYDEVRAGA) and the previously described peptide LLRRYNVAKV could induce significant peptide-specific CD8^+^ T cell responses in the IFN-γ ELISPOT assay [21]. These assays demonstrated that the peptide FMACSPVAL, derived from SOX11, yielded the strongest results and could serve as an effective peptide for activating tumor-specific CD8^+^ T cell responses.

All the positive peptide-specific CD8^+^ T cell responses for FMACSPVAL, LLRRYNVAKV, LMFDLSLNF, and SLYDEVRAG are shown in Figure 4. The spots were counted and evaluated (Table 3). The results were considered positive when the spot count was at least twice as high as that of the negative control. Mean values were calculated for each group; error bars indicate SD.

### 2.6. Recognition of Different Peptides by T Cells from Different Healthy Donors

**Figure 5:**
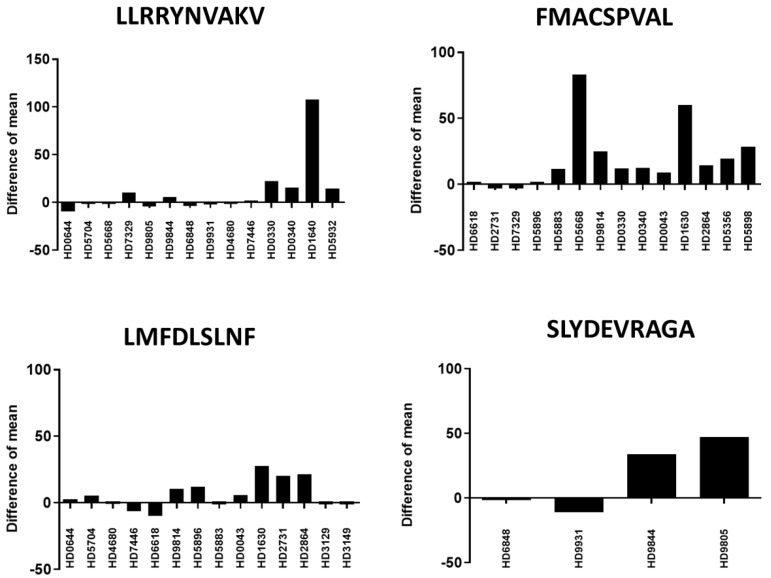
IFN-γ ELISPOT assay showing the peptide-specific activation of T cells. Cells were evaluated in triplicate for each ELISPOT assay, and means were calculated. This figure shows the net mean ELISPOT counts reduced by mean background spot numbers for four different peptides.

An IFN-γ ELISPOT assay was performed to show the activated peptide-specific T cells induced by different peptides from different healthy donor samples. Figure 5 shows the net mean ELISPOT counts reduced by mean background spot numbers (ELISPOT counts of primed T cells co-cultured with peptide-loaded T2 cells-primed T cells co-cultured with unloaded T2 cells) for different healthy donors. The results showed that the FMACSPVAL-specific CD8^+^ T cells could induce more IFN-γ-secreting cells when co-cultured with peptide-loaded T2 cells than when co-cultured with unloaded T2 cells in more than 70% of the healthy donor samples, while the LLRRYNVAKV-specific CD8^+^ T cells induced more IFN-γ-secreting cells under the abovementioned conditions in less than 50% of the healthy donor samples (Figure 5).

### 2.7. Evaluation of Cytokine Production and Cytotoxicity of Peptide-Specific CD8^+^ T Cells

**Figure 6:**
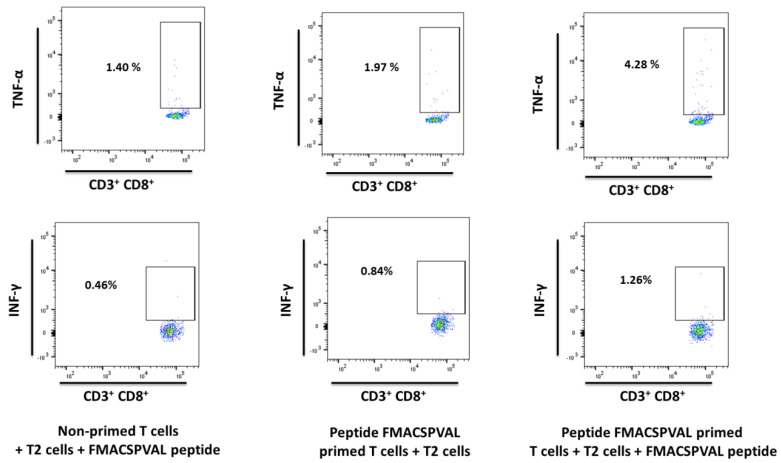
Intracellular flow cytometry staining (HD 3026, E:T ratio 1:10) primed with peptide FMACSPVAL. The ability of FMACSPVAL-specific CD8^+^ T cells to secrete the cytokines IFN-γ and TNF-α after stimulation was evaluated by intracellular flow cytometry staining. A non-primed T cell group and unloaded T2 cell group served as negative controls. Exemplary flow cytometry plots of positive responses are shown. Percentages of IFN-γ^+^ and TNF-α^+^ cells after peptide-stimulation were analyzed and compared with negative control samples. T cells were gated on CD3^+^ CD8^+^ cells.

In order to evaluate the ability of FMACSPVAL-specific CD8^+^ T cells to secrete the cytokines IFN-γ and TNF-α after stimulation, intracellular flow cytometry staining was performed. FMACSPVAL-specific CD8^+^ T cells were incubated with FMACSPVAL-loaded T2 cells for 5 h before intracellular cytokine staining. A non-primed T cell group and an unloaded T2-cell group served as negative controls. The level of IFN-γ production changed slightly. In contrast, TNF-α production was significantly higher in the FMACSPVAL-specific CD8^+^ T cells incubated with FMACSPVAL-loaded T2 cells when compared with the non-primed T cells incubated with FMACSPVAL-loaded T2 cells group and the FMACSPVAL-specific CD8^+^ T cells incubated with unloaded T2 cells group (Figure 6).

**Figure 7:**
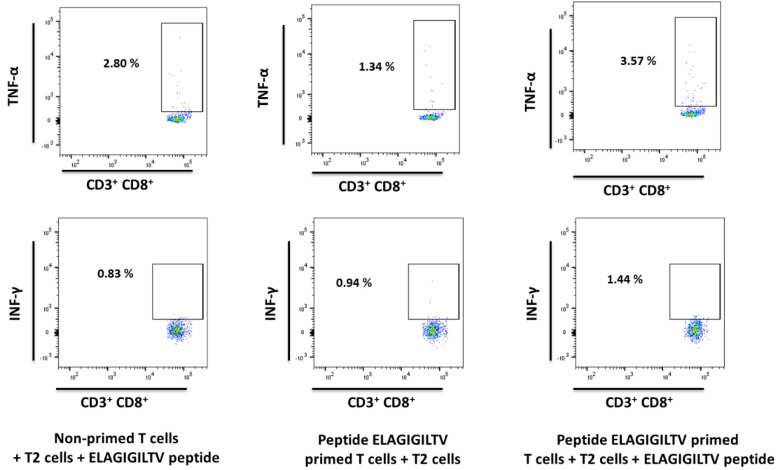
Intracellular flow cytometry staining (HD 3026, E:T ratio 1:10) primed with peptide ELAGIGILTV. The ability of ELAGIGILTV-specific CD8^+^ T cells to secrete the cytokines IFN-γ and TNF-α after stimulation was evaluated by intracellular flow cytometry staining. Exemplary flow cytometry plots of positive responses are shown. T cells were gated on CD3^+^ CD8^+^ cells.

**Figure 8:**
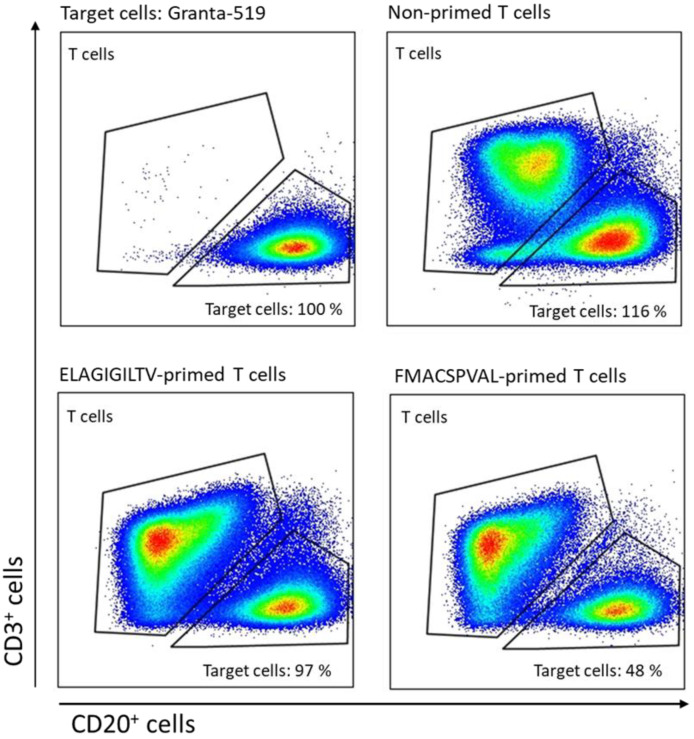
Cytotoxic capability of SOX11-specific T cells. FMACSPVAL-primed T cells were able to kill 52% of target SOX11-expressing cells. Non-primed T cells served as a negative control. Target cells were co-cultured with T cells for 24 h before staining and detection via flow cytometry.

ELAGIGILTV-specific CD8^+^ T cells were used as a positive control to measure the IFN-γ and TNF-α secretion ability. The level of IFN-γ production and the level of TNF-α production by ELAGIGILTV-specific CD8^+^ T cells co-cultured with ELAGIGILTV-loaded T2 cells increased compared to the negative control (Figure 7). To determine whether peptide-specific T cells affected SOX11-positive target cells, we performed a cytotoxicity assay using Granta-519 cells as a target (Figure 8). Granta-519 cells were killed by FMACSPVAL-primed T cells; however, they were not killed by non-primed T cells. Taken together, the peptide FMACSPVAL seemed to be presented by tumor cell lines and was recognized by T cells specific to the HLA-A*0201-restricted FMACSPVAL peptide epitope.

## 3. Discussion

As great progress has been made in the T cellular immunotherapy of refractory malignancies, therapeutic strategies for glioma are also becoming a new focus. Based on the expression profile, tumor antigens can be roughly divided into three categories: tumor-associated antigens, which have a low expression in normal cells and are overexpressed in tumor cells; cancer germline antigens, which are expressed in tumor cells of different histological origins but silent in normal adult tissues; and tumor-specific antigens, with expression only in tumor cells and not in normal healthy cells [25]. Tumor-associated antigens usually serve as targets for T cells. The selection of an optimal target antigen is one of the most crucial steps in adoptive T cell therapy (ACT). Targeting non-mutated antigens that are overexpressed on tumors but also expressed on normal cells has led to severe off-tumor, on-target toxicity in patients [26]. Appropriate antigens for ACT should be expressed only on tumor cells or non-essential healthy tissue. The transcription factor SOX11 is expressed during embryogenesis but largely absent in most adult differentiated tissues. SOX11 regulates progenitor and stem cell behavior and often acts together with the other two SOXC group members, SOX4 and SOX12, in regulating developmental processes, including neurogenesis and skeletogenesis. The dysregulation of SOX11 has been implicated in a number of diseases, including neurodevelopmental disorders and osteoarthritis, and a wide variety of cancers [27].

Malignant glioma is known to be genetically heterogenous, with a variety of antigen profiles [28]. Difficulty in the identification of ideal tumor antigens for immunotherapy and the immune evasion mechanisms of tumor cells may induce malignant gliomas resistant to T cell responses. So far, several glioma-associated antigens have been identified, but the antigens most suitable for activating the tumor-specific T cell response are still under investigation [7]. Some of these are cell-surface antigens that can be targeted by CAR T cell therapies, and others are derived from intracellular proteins that can be targeted by vaccines or T cell-receptor-transduced T cell therapy [22]. Recently, glioma-specific antigens assessed in preclinical studies have shown potential anti-glioma effects, such as epidermal growth factor receptor variant III (EGFRvIII) [29], human epidermal growth factor receptor 2 (HER2) [30], and erythropoietin-producing hepatocellular carcinoma A2 (EphA2) [31]. In this study, we investigated the suitability of the transcription factor SOX11, a known glioma-associated antigen, as another promising target antigen for immunotherapy.

At the beginning of this study, we used the state-of-the-art prediction server at that time (Table 1), and at the end of the study we checked the selected peptides again with the algorithm (NetMHCpan 4.1) currently considered as the best prediction server (Table 4). According to the recommended readout “Percentile Rank Eluted Ligands” (EL %rank), a value under 0.5 indicates a strong binder, a value under 2.0 a weak binder, and other peptides are non-binders. We could confirm that the peptide with the highest score predicted using NetMHCpan 4.1 also achieved the best results in the experimental examinations. Two further peptides that were analyzed more precisely could also be predicted as binders. All peptides that showed no peptide-specific CD8^+^ T cell responses in the IFN-γ ELISPOT assay were correctly classified by the latest predictor as non-binders.

An exception was the peptide KMLKDSEKI, which was predicted as a strong binder by NetMHCpan 4.1. This result could not be confirmed in an experimental setup. Moreover, the previously described peptide LLRRYNVAKV was also classified by NetMHCpan 4.1 as a non-binder [21]. In conclusion, the tools were able to make an initial prediction, but an experimental conformation of these results was still needed. The prediction tools cannot entirely replace the further examination of promising peptides, but our latest results confirmed an improvement in the prediction ability of the algorithm.

The ex vivo expansion and manipulation of SOX11-specific T cells is a crucial step for developing effective cancer immunotherapy. However, various protocols exist with differing culture conditions, including stimulation, activation, and supplementation with cytokines, to generate these specific T cells (Table 5). We compared two different manufacturing methods that provided functional tumor-specific T cells and had individual advantages and disadvantages. 

The short method could achieve the faster generation of SOX11-specific CD8^+^ T cells within 12 days. DCs were generated without the additional presorting of cells and were cultured together with PBMCs. Therefore, a smaller amount of frozen PBMCs could be used. This allowed us to repeat experiments with cells from the same healthy donor, and more peptides could be analyzed with PBMCs from the same donor. It was more useful for the fast ex vivo screening of many candidate antigens.

For the long method, fresh PBMCs were needed to obtain mature DCs. The complete duration of the process for generating the peptide-specific T cells was 21 days, because the DCs needed to be matured separately before stimulation. This method had a higher efficiency for generating the SOX11-specific CD8^+^ T cells (Table 2). This was perhaps in part because the peptide-pulsed mature DCs were added to cultured T cells for a second stimulation, whereas for the short method only one cycle of stimulation with peptides was required to provide peptide-specific T cells. Additionally, for the long method, better results could be achieved when a second stimulation was added. The longer method seemed to generate a higher proportion of SOX11-specific CD8^+^ T cells and may have generated a higher number of SOX11-specific T cells that were of a higher quality and functionality. In vivo studies are necessary to confirm the comparability or even the superiority of one of these protocols.

Over the past two decades, DC-based immunotherapy has offered a promising approach for improving the outcomes of glioma patients. Successful DC-based brain tumor immunotherapy has been reported in animal models and patients by Yamanaka et al. [32], Yu et al. [33], and Kikuchi et al. [34]. These authors also found that DC vaccination appears to be safe and not associated with autoimmunity. DC-based immunotherapy can be divided into two methods: direct immunization with antigen-pulsed DCs and the adoptive transfer of in vitro expanded cytotoxic T lymphocytes following stimulations with antigen-pulsed DCs [35]. Since a variety of immunological defects have been found, including decreased T cell numbers and impaired T cell responsiveness [36], the adoptive transfer of in vitro activated and expanded tumor-specific CD8^+^ T cells seems to be the most appropriate choice for treating glioma patients. A great challenge for adoptive T cell therapy is the identification of HLA class-I restricted CD8^+^ T cell epitopes derived from glioma-associated antigens. The DNA mutation or gene translocation of cancer-related genes can result in the expression of altered proteins containing amino acid residues that differ from normal ones [37]. De la Rocha et al. [19] showed that the aberrant expression of members of the SOX family is relevant to the development of CNS malignant tumors. Weigle et al. [20] showed that SOX11 is abundantly and specifically overexpressed in GBM. The expression of SOX11 is probably reactivated during tumorigenesis. Schmitz et al. [21] reported the identification of an immunogenic CD8^+^ T cell epitope derived from SOX11, which emphasized the suitability of this protein for a T cell-based immunotherapy for GBM patients. Our study extended the previous work of Schmitz et al. We compared the results of two manufacturing protocols by screening promising peptides and described for the first time the new attractive peptide FMACSPVAL, which had a higher efficiency for generating SOX11-specific CD8^+^ T cells than the peptide LLRRYNVAKV previously reported by Schmitz et al. [21]. Thomas Trolle et al. showed that all HLAs have a peptide length preference for 9 mer peptides [38]. HLA-A*0201 has a strong preference for 9 mers, whereas HLA-A*0101 has an increased preference for 10 mers. In our study, we selected the nine peptides with the highest binding affinity by comparing the results of different online algorithms. All of them had a length of 9–10 amino acids. The most promising peptide, FMACSPVAL, is a 9 mer peptide, while the previously reported peptide LLRRYNVAKV, considered a potential HLA-A*0201 ligand, is a 10 mer peptide [21]. Accordingly, our results showed that the peptide FMACSPVAL had a higher efficiency for generating SOX11-specific CD8^+^ T cells than the peptide LLRRYNVAKV. Moreover, we confirmed the results of the INF-γ ELISPOT assay for FMACSPVAL-specific CD8^+^ T cells by detecting the secretion of TNF-α after stimulation by intracellular cytokine staining. This will broaden the therapeutic options for glioma patients. Our future investigations will focus on the potential use of FMACSPVAL-specific CD8^+^ T cell immunotherapy in rodent models of SOX11-overexpressing CNS malignancies.

## 4. Materials and Methods

### 4.1. Blood Samples from Healthy Donors

Buffy coats from voluntary healthy-donor (HD) blood samples were obtained from the blood bank of the Institute of Clinical Transfusion Medicine and Cell Therapy (IKTZ, Heidelberg, Germany). All subjects provided informed consent for inclusion before they participated in the study. The study was conducted in accordance with the Declaration of Helsinki, and the protocol was approved by the Ethics Committee of Heidelberg (S 254/2016). PBMCs were isolated from peripheral blood samples by Ficoll-Hypaque gradient purification (Biochrom, Berlin, Germany). Cells were cryopreserved in 90% heat-inactivated fetal bovine serum (FBS, Gibco^®^, Grand Island, NY, USA) supplemented with 10% dimethyl sulfoxide (DMSO, Sigma-Aldrich, St. Louis, MO, USA) and stored in liquid nitrogen.

### 4.2. Cell Line

The T2 cell line, which was used to provide antigen-presenting cells, was obtained from the American Type Culture Collection (Manassas, VA, USA). It is an HLA-A*0201-expressing T-B lymphoblastoid hybrid cell line, which was cultured in a medium consisting of RPMI 1640, 10 U/mL penicillin/0.1 mg/ml streptomycin, 2 mM L-glutamine, and 10% heat-inactivated FBS (all from PAA Laboratories, Pasching, Austria) at 37 °C, 5% CO_2_.

### 4.3. Epitope Prediction and Peptide Synthesis

Potential HLA-A*0201 ligands were selected from the amino acid sequence of SOX11. The prediction algorithms and NetMHC 4.0, NetMHCpan-3.0, NetMHCcons 1.1, NetMHC 3.4, NetMHCpan 2.8, NetMHCpan4.1, NetMHCcons pickpocket 1.1, IEDB recommended, IEDB consensus, IEDB smmpmbec, IEDB smm, and SYFPEITHI were simultaneously queried with the tool MHCcombine (https://mhccombine.dkfz.de/mhccombine/) for 9 mer and 10 mer HLA-A*0201-binding peptides. The binding cutoffs for the different prediction algorithms defined in Bonsack et al. (2019) [23] were applied. The MART-1 analogue peptide (ELAGIGILTV) and 10 SOX-11 peptides were synthesized by the Peptide Synthesis Unit of the German Cancer Research Center Heidelberg (Heidelberg, Germany).

### 4.4. HLA Typing

HLA-A*0201 typing was performed as previously reported by staining PBMCs with anti-human HLA-A*0201 FITC conjugated antibodies (Biolegend, San Diego, CA, USA) for 30 min at 4 °C in the dark [39]. Data acquisition was performed using a BDTM LSR II flow cytometer, and data were analyzed using FlowJo software (Becton Dickinson, New York, NY, USA).

### 4.5. In Vitro Generation of Peptide-Specific CD8^+^ T Cells with Long Method

PBMCs were divided into two parts. One part was frozen for later use. The other part was used for generating DCs. Therefore, PBMCs were cultured in a sterile flask to let monocytes adhere at 37 °C, 5% CO_2_ for 1 h. The non-adherent lymphocytes were collected and frozen at −80 °C. The adherent monocytes were left in the flask and were then incubated with a medium consisting of RPMI1640, 2 mM L-glutamine, 10 U/mL penicillin/0.1 mg/ml streptomycin, 5% heat-inactivated human serum, 800 U/mL human GM-CSF, and 500 U/mL IL-4 for 5–6 days. Afterwards, differentiation into mature DCs was induced by adding 800 U/mL human GM-CSF, 500 U/mL IL-4, 10 ng/mL TNF-a, 1 μg/mL PGE2, 1000 U/mL IL-6, and 1000 U/mL IL-1ß for 2 days at 37 °C, 5% CO2. To generate peptide-specific T cells, mature DCs were preincubated for 3 h with 10 µg/mL of the respective peptide in serum-free medium. The cryopreserved lymphocytes were thawed and incubated together with peptide-pulsed mature DCs (DCs/lymphocytes ratio 1:50) in a T cell medium consisting of RPMI 1640, 2 mM L-glutamine, 10 U/mL penicillin + 0.1 mg/mL streptomycin, 5% heat-inactivated human serum, and 10 ng/mL IL-7 for 7 days. Every two days, 50 U/mL IL-2 was added. At the same time, cryopreserved PBMCs were thawed to generate new peptide-pulsed mature DCs using the same method for restimulation. After 7 days of culture, freshly produced peptide-pulsed mature DCs were added to the cultured T cells.

### 4.6. In Vitro Generation of Peptide-Specific CD8^+^ T Cells with Short Method

The generation of peptide-specific CD8^+^ T cells was carried out as previously described by Lissina et al., 2016 [40]. Cryopreserved PBMCs were thawed and incubated with a medium consisting of RPMI1640, 10 U/mL penicillin + 0.1 mg/mL streptomycin, 800 U/mL human GM-CSF, and 500 U/mL IL-4 for 1 day. On the next day, 10 ng/mL TNF-a, 1 μg/mL PGE2, 1000 U/mL IL-1ß,10 ng/mL IL-7, and 10 µg/mL of the respective peptides were added. The medium with RPMI1640, 10 U/mL penicillin + 0.1 mg/ml streptomycin, 10% heat-inactivated FBS, 5 ng/mL IL-7, 25 ng/mL IL-15, and 100 U/mL IL-2 was replaced every 3 days. After 8 days, the cells were tested for their ability to secrete INF-γ by an ELISPOT assay.

### 4.7. HLA-A*02-Binding Affinity Assay

To determine the binding affinity of the selected peptides to the HLA-A*0201 molecules, the peptide-induced expression of HLA-A*0201 on T2 cells was measured by flow cytometry. The T2 cells were incubated for 18 h at 26 °C in 5% CO_2_ with 10 µg/mL of the respective peptides and 3 µg/mL human β2 microglobulin (Thermo Fisher Scientific, Waltham, MA, USA). The temperature was raised to 37 °C for 2 h. The T2 cells were harvested and stained with anti-human HLA-A*0201 FITC conjugated antibodies (Biolegend, San Diego, CA, USA) for 30 min at 4 °C. Samples were measured using a BDTM LSR II flow cytometer and analyzed using FlowJo software (Becton Dickinson, New York, NY, USA). The percent mean fluorescence index (% MFI) increase of the HLA-A*0201 molecules was evaluated using the following equation: % MFI increase = ((MFI with peptide − MFI without peptide))/(MFI without peptide) × 100.

### 4.8. IFN-γ ELISPOT Assay

The IFN-γ ELISPOT assay was performed as described previously [41]. T2 cells were preincubated for 2 h with 10 µg/mL of the respective peptides to obtain peptide-loaded T2 cells. T cells from each group were purified to obtain CD8^+^ T cells with immunomagnetic MACS beads (MACS system; Miltenyi Biotec, Bergisch Gladbach, Germany) following the manufacturer’s instructions. The CD8^+^ T cells were incubated as effector cells (2 × 10^4^/well) with peptide-loaded T2 cells as targets (1 × 10^5^/well) at an effector-cells-to-targeT cells ratio of 1:5 in anti–IFN-γ Ab (Mabtech, Nacka, Sweden)-precoated 96-well nitrocellulose plates (Millipore, Eschborn, Germany) with a final volume of 200 µL culture medium for 48 h. Biotinylated anti-cytokine antibodies (Mabtech, Nacka, Sweden) were added to each well for 1.5 h following 1 h incubation with avidin alkaline phosphatase (Sigma-Aldrich, St. Louis, MO, USA). Afterwards, 5-bromo-4-chloro-3-indolyl phosphate/Nitro blue tetrazolium substrate (Sigma-Aldrich, St. Louis, MO, USA) was added, and the spots emerged after 5–10 min. The reaction was stopped by washing the plates with water. A spot count was performed using an ELISPOT reader (CTL-Europe GmbH, Bonn, Germany).

### 4.9. Intracellular Cytokine Staining

The cytokines IFN-γ and TNF-α were detected by intracellular cytokine staining and flow cytometry. Peptide-specific T cells were co-cultured with corresponding peptide-loaded T2 cells in the presence of Brefeldin A (Biolegend, San Diego, CA, USA) for 5 h at an effector-to-target ratio of 1:10. Non-primed T cells and unloaded T2 cells served as negative controls. After the NEAR-IR (Invitrogen, Thermo Fisher Scientific, Waltham, MA, USA) and surface marker staining, cells were fixed and permeabilized for 30 min at room temperature (RT) in the dark using the FoxP3 staining buffer kit (Miltenyi Biotec, Bergisch Gladbach, Germany) with subsequent washing and resuspension in FoxP3 Perm Buffer (Miltenyi Biotec, Bergisch Gladbach, Germany) for 15 min at RT. Anti-IFN-γ-APC (Biolegend, San Diego, CA, USA) and anti-TNF-α-BV421 (BD Biosciences, San Diego, CA, USA) were applied to detect the levels of IFN-γ and TNF-α, respectively (RT, 30 min). For surface marker staining, the following antibodies were used: CD3 PE (Biolegend, San Diego, CA, USA); CD8 PerCP (Biolegend, San Diego, CA, USA); CD197 (CCR7) PE-Cy7 (eBioscience, San Diego, CA, USA); and CD45RA Alexa Fluor 700 (Biolegend, San Diego, CA, USA). Finally, cells were resuspended in 500 μL FACS Buffer (Miltenyi Biotec, Bergisch Gladbach, Germany). Samples were measured with a BDTM LSR II flow cytometer and analyzed using FlowJo software (Becton Dickinson, New York, NY, USA).

### 4.10. Cytotoxic Capacitiy of SOX11-Specific CAR T Cells

Granta-519 cells were irradiated (100Gy) and co-cultured with FMACSPVAL-primed T cells, ELAGIGILTV-primed T cells, or non-primed T cells as a negative control. Cells were co-cultured at an effector:target ratio of 2:1 for 24 h. Samples were harvested and spiked with a defined number of polystyrene beads (Life Technologies Corporation, Eugene, OR, USA) to quantify cell numbers by flow cytometry. Cytotoxicity was assessed by determining the number of viable Ganta-519 cells in the presence of T cells relative to that in the absence of T cells.

### 4.11. Statistical Analysis

Statistical analysis was performed using Excel (Microsoft, Redmond, WA, USA). Statistical significance was calculated using a paired-sample *t*-test and is represented as * for *p* values < 0.05, which were considered statistically significant. Graphs and tables were designed using Excel or Prism 9.0 (GraphPad Software, San Diego, CA, USA). Mean values were calculated for each group; error bars indicate standard deviation (SD).

## 5. Conclusions

SOX11 was shown to be overexpressed in GBM, making it a promising target antigen for immunotherapy. We selected potential HLA-A*0201 ligands from the amino acid sequence of SOX11 by in silico prediction and compared two different methods for generating peptide-specific CD8^+^ T cells. After the comparison of the ten selected peptides, including a previously published peptide, we identified the new attractive peptide FMACSPVAL, which had the highest in silico prediction score, the highest binding affinity to the HLA-A*0201 molecules according to the peptide-binding assay on T2 cells, and the highest efficiency for generating SOX11-specific CD8^+^ T cells according to the IFN-γ ELISPOT assay. Therefore, the SOX11-derived peptide FMACSPVAL may serve as an epitope for a T cell-based immunotherapy for glioma.

## Figures and Tables

**Table 1:** HLA-A*0201-binding peptides derived from SOX11 as defined by in silico prediction.

Peptide Sequence	Position ^a^	Length ^b^	NetMHC-4.0 (nM)	Predicted Binding Level ^c^	Number of Other Queried Algorithms That Predicted Binding ^d^
FMACSPVAL	26–34	9	6.02	strong	10
EFMACSPVAL	25–34	10	34.53	strong	1
WLEANFSDLV	429–438	10	79.27	weak	10
FMACSPVALD	26–35	10	117.95	weak	9
SLYDEVRAGA	292–301	10	155.74	weak	10
LMFDLSLNF	361–369	9	250.95	weak	9
MIAGDWLEA	424–432	9	258.82	weak	10
LMFDLSLNFS	361–370	10	261.92	weak	10
KMLKDSEKI	88–96	9	316.75	weak	10
LLRRYNVAKV	266–275	10	1469.73	none	10
Positive control: ELAGIGILTV	MART-1 26–35	10	253.92	weak	10

^a^ The position of the peptide in the amino acid sequence. ^b^ Number of amino acids. ^c^ Binding level cutoffs as determined by NetMHC-4.0. ^d^ Binding cutoffs as defined in [23].

**Table 2:** In vitro generation of CD8^+^ T cells specifically recognizing the SOX11-derived peptides.

Peptide Sequence	ELISPOT ^a^ ^(long method)^	ELISPOT ^a^ ^(short method)^	ELISPOT ^a^ ^(short method + long method)^	ICS (TNF-α) ^b^ ^(long method)^	ICS (IFN-γ) ^c^ ^(long method)^
ELAGIGILTV	3:4	8:18	11:22	4:10	2:10
FMACSPVAL	6:14	3:18	9:32	1:10	1:10
LLRRYNVAKV	1:14	1:8	2:22	-	-
LMFDLSLNF	1:14	1:8	2:22	-	-
SLYDEVRAGA	2:4	1:8	3:12	-	-
EFMACSPVAL	0:3	1:8	1:11	-	-
FMACSPVALD	0:3	1:8	1:11	-	-
MIAGDWLEA	0:3	0:8	0:11	-	-
WLEANFSDLV	0:3	0:8	0:11	-	-
KMLKDSEKI	0:3	0:8	0:11	-	-
LMFDLSLNFS	0:3	0:8	0:11	-	-

^a^ Ratio of number of healthy donors presenting positive antigen-specific responses to all healthy donors, with detection by ELISPOT assay. ^b^ Ratio of number of healthy donors presenting positive antigen-specific responses to all healthy donors, with detection by intracellular cytokine staining and analysis for intracellular expression of TNF-α. ^c^ Ratio of number of healthy donors presenting positive antigen-specific responses to all healthy donors, with detection by intracellular cytokine staining and analysis for intracellular expression of IFN-γ.

**Table 3:** Summary of peptide-specific T cell responses from different healthy donors.

Peptide Sequence	Healthy Donor Sample (HD)	Unloaded T2 Cells	Peptide-Loaded T2 Cells	*p*-Value
LLRRYNVAKV	HD 1640	7.75 ± 2.00	115.25 ± 13.71	0.0001
FMACSPVAL	HD 0340	7.60 ± 3.21	19.00 ± 6.40	0.0076
FMACSPVAL	HD 5668	12.40 ± 5.21	95.20 ± 28.20	0.0002
FMACSPVAL	HD 5883	8.67 ± 3.50	20.00 ± 7.00	0.0400
FMACSPVAL	HD 5356	2.40 ± 2.90	22.00 ± 6.00	0.0002
FMACSPVAL	HD 5898	9.00 ± 2.70	37.40 ± 7.80	0.0001
FMACSPVAL	HD 1630	15.60 ± 8.00	75.60 ± 17.70	0.0001
LMFDLSLNF	HD 5896	9.33 ± 4.00	21.33 ± 3.11	0.0148
SLYDEVRAG	HD 9805	11.00 ± 1.20	51.80 ± 31.80	0.0210
SLYDEVRAG	HD 9844	25.60 ± 9.21	59.60 ± 22.00	0.0128

**Table 4:** HLA-A*0201-binding peptides derived from SOX-11 as defined by NetMHCpan 4.1.

Position Start	Region	Length	Sequence	NetMHCpan 4.1 (EL %rank)	NetMHCpan 4.1 (nM)
26	26–34	9	FMACSPVAL	0.309	4.45
25	25–34	10	EFMACSPVAL	7.239	152.99
429	429–438	10	WLEANFSDLV	4.360	48.37
26	26–35	10	FMACSPVALD	7.021	166.12
292	292–301	10	SLYDEVRAGA	0.342	91.27
361	361–369	9	LMFDLSLNF	1.230	156.17
424	424–432	9	MIAGDWLEA	2.829	310.33
361	361–370	10	LMFDLSLNFS	6.528	158.03
88	88–96	9	KMLKDSEKI	0.471	234.52
266	266–275	10	LLRRYNVAKV	12.828	2439.73

**Table 5:** Main differences between the two methods used in this study to expand specific T cells.

	Short Method	Long Method
Cells	Cryopreserved PBMCs	Fresh PBMCs
Cell number	6 × 10^7^	4 × 10^8^
Incubation time	12 days	21 days
Stimulation	One-week stimulation	Two-week stimulation
Activation conditions	DCs and PBMCs are cultured together	DCs are matured separately
Cytokine cocktail 1	800 U/mL human GM-CSF, 500 U/mL IL-4	2 mM L-glutamine, 5% human serum, 800 U/mL human GM-CSF, 500 U/mL IL-4
Cytokine cocktail 2	10 ng/mL TNF-α, 1 μg/mL PGE2, 1000 U/mL IL-1ß, and 10 ng/mL IL-7	10 ng/mL TNF-α, 1 μg/mL PGE2, 1000 U/mL IL-6, and 1000 U/mL IL-1ß
Incubation medium of peptide-specific T cells	RPMI1640, 10% FBS, 5 ng/mL IL-7, 25 ng/mL IL-15, 100 U/mL IL-2	RPMI 1640, 2 mM L-glutamine, 5% human serum, 10 ng/mL IL-7, 50 U/mL IL-2

## Data Availability

Data are on file with our lab and available upon request.

## References

[1] Herholz K. (2017). Brain Tumors: An Update on Clinical PET Research in Gliomas. Semin. Nucl. Med..

[2] Zhang N., Zhang L., Qiu B., Meng L., Wang X., Hou B.L. (2012). Correlation of volume transfer coefficient Ktrans with histopathologic grades of gliomas. J. Magn. Reson. Imaging.

[3] Ostrom Q.T., Bauchet L., Davis F.G., Deltour I., Fisher J.L., Langer C.E., Pekmezci M., Schwartzbaum J.A., Turner M.C., Walsh K.M. (2014). The epidemiology of glioma in adults: A “state of the science” review. Neuro Oncol..

[4] Oberoi R.K., Parrish K.E., Sio T.T., Mittapalli R.K., Elmquist W.F., Sarkaria J.N. (2016). Strategies to improve delivery of anticancer drugs across the blood-brain barrier to treat glioblastoma. Neuro Oncol..

[5] Xu S., Tang L., Li X., Fan F., Liu Z. (2020). Immunotherapy for glioma: Current management and future application. Cancer Lett..

[6] Chung D.S., Shin H.J., Hong Y.K. (2014). A new hope in immunotherapy for malignant gliomas: Adoptive T cell transfer therapy. J. Immunol. Res..

[7] Leen A.M., Rooney C.M., Foster A.E. (2007). Improving T cell therapy for cancer. Annu. Rev. Immunol..

[8] Wheeler C.J., Black K.L., Liu G., Mazer M., Zhang X.X., Pepkowitz S., Goldfinger D., Ng H., Irvin D., Yu J.S. (2008). Vaccination elicits correlated immune and clinical responses in glioblastoma multiforme patients. Cancer Res..

[9] Pevny L., Placzek M. (2005). SOX genes and neural progenitor identity. Curr. Opin. Neurobiol..

[10] Mitchell D.A., Fecci P.E., Sampson J.H. (2003). Adoptive immunotherapy for malignant glioma. Cancer J..

[11] Schuler M.M., Nastke M.D., Stevanovikc S. (2007). SYFPEITHI: Database for searching and T cell epitope prediction. Methods Mol. Biol..

[12] Reynisson B., Alvarez B., Paul S., Peters B., Nielsen M. (2020). NetMHCpan-4.1 and NetMHCIIpan-4.0: Improved predictions of MHC antigen presentation by concurrent motif deconvolution and integration of MS MHC eluted ligand data. Nucleic Acids Res..

[13] Jurtz V., Paul S., Andreatta M., Marcatili P., Peters B., Nielsen M. (2017). NetMHCpan-4.0: Improved peptide–MHC class I interaction predictions integrating eluted ligand and peptide binding affinity data. J. Immunol..

[14] Nielsen M., Andreatta M. (2016). NetMHCpan-3.0; improved prediction of binding to MHC class I molecules integrating information from multiple receptor and peptide length datasets. Genome Med..

[15] Hoof I., Peters B., Sidney J., Pedersen L.E., Sette A., Lund O., Buus S., Nielsen M. (2009). NetMHCpan, a method for MHC class I binding prediction beyond humans. Immunogenetics.

[16] Andreatta M., Nielsen M. (2016). Gapped sequence alignment using artificial neural networks: Application to the MHC class I system. Bioinformatics.

[17] Nielsen M., Lundegaard C., Worning P., Lauemøller S.L., Lamberth K., Buus S., Brunak S., Lund O. (2003). Reliable prediction of T cell epitopes using neural networks with novel sequence representations. Protein Sci..

[18] Sarkar A., Hochedlinger K. (2013). The sox family of transcription factors: Versatile regulators of stem and progenitor cell fate. Cell Stem Cell.

[19] de la Rocha A.M., Sampron N., Alonso M.M., Matheu A. (2014). Role of SOX family of transcription factors in central nervous system tumors. Am. J. Cancer Res..

[20] Weigle B., Ebner R., Temme A., Schwind S., Schmitz M., Kiessling A., Rieger M.A., Schackert G., Schackert H.K., Rieber E.P. (2005). Highly specific overexpression of the transcription factor SOX11 in human malignant gliomas. Oncol. Rep..

[21] Schmitz M., Wehner R., Stevanovic S., Kiessling A., Rieger M.A., Temme A., Bachmann M., Rieber E.P., Weigle B. (2007). Identification of a naturally processed T cell epitope derived from the glioma-associated protein SOX11. Cancer Lett..

[22] Nejo T., Yamamichi A., Almeida N.D., Goretsky Y.E., Okada H. (2020). Tumor antigens in glioma. Semin. Immunol..

[23] Bonsack M., Hoppe S., Winter J., Tichy D., Zeller C., Küpper M.D., Schitter E.C., Blatnik R., Riemer A.B. (2019). Performance evaluation of MHC class-I binding prediction tools based on an experimentally validated MHC–peptide binding data set. Cancer Immunol. Res..

[24] Valmori D., Fonteneau J.-F., Lizana C.M., Gervois N., Liénard D., Rimoldi D., Jongeneel V., Jotereau F., Cerottini J.-C., Romero P. (1998). Enhanced generation of specific tumor-reactive CTL in vitro by selected Melan-A/MART-1 immunodominant peptide analogues. J. Immunol..

[25] Bonaventura P., Shekarian T., Alcazer V., Valladeau-Guilemond J., Valsesia-Wittmann S., Amigorena S., Caux C., Depil S. (2019). Cold Tumors: A Therapeutic Challenge for Immunotherapy. Front. Immunol..

[26] Lamers C.H., Sleijfer S., van Steenbergen S., van Elzakker P., van Krimpen B., Groot C., Vulto A., den Bakker M., Oosterwijk E., Debets R. (2013). Treatment of metastatic renal cell carcinoma with CAIX CAR-engineered T cells: Clinical evaluation and management of on-target toxicity. Mol. Ther..

[27] Tsang S.M., Oliemuller E., Howard B.A. (2020). In Regulatory roles for SOX11 in development, stem cells and cancer. Semin. Cancer Biol..

[28] Zhang J.G., Eguchi J., Kruse C.A., Gomez G.G., Fakhrai H., Schroter S., Ma W., Hoa N., Minev B., Delgado C. (2007). Antigenic profiling of glioma cells to generate allogeneic vaccines or dendritic cell-based therapeutics. Clin. Cancer Res..

[29] Bullain S.S., Sahin A., Szentirmai O., Sanchez C., Lin N., Baratta E., Waterman P., Weissleder R., Mulligan R.C., Carter B.S. (2009). Genetically engineered T cells to target EGFRvIII expressing glioblastoma. J. Neurooncol..

[30] Ahmed N., Salsman V.S., Kew Y., Shaffer D., Powell S., Zhang Y.J., Grossman R.G., Heslop H.E., Gottschalk S. (2010). HER2-specific T cells target primary glioblastoma stem cells and induce regression of autologous experimental tumors. Clin. Cancer Res..

[31] Chow K.K., Naik S., Kakarla S., Brawley V.S., Shaffer D.R., Yi Z., Rainusso N., Wu M.-F., Liu H., Kew Y. (2013). T cells redirected to EphA2 for the immunotherapy of glioblastoma. Mol. Ther..

[32] Yamanaka R., Abe T., Yajima N., Tsuchiya N., Homma J., Kobayashi T., Narita M., Takahashi M., Tanaka R. (2003). Vaccination of recurrent glioma patients with tumour lysate-pulsed dendritic cells elicits immune responses: Results of a clinical phase I/II trial. Br. J. Cancer.

[33] Yu J.S., Wheeler C.J., Zeltzer P.M., Ying H., Finger D., Lee P., Yong W., Incardona F., Thompson R., Riedinger M. (2001). Vaccination of malignant glioma patients with peptide-pulsed dendritic cells elicits systemic cytotoxicity and intracranial T cell infiltration. Cancer Res..

[34] Kikuchi T., Akasaki Y., Irie M., Homma S., Abe T., Ohno T. (2001). Results of a phase I clinical trial of vaccination of glioma patients with fusions of dendritic and glioma cells. Cancer Immunol. Immunother..

[35] Wu A.-h., Xiao J., Anker L., Hall W.A., Gregerson D.S., Cavenee W.K., Chen W., Low W.C. (2006). Identification of EGFRvIII-derived CTL epitopes restricted by HLA A0201 for dendritic cell based immunotherapy of gliomas. J. Neurooncol..

[36] Morford L.A., Dix A.R., Brooks W.H., Roszman T.L. (1999). Apoptotic elimination of peripheral T lymphocytes in patients with primary intracranial tumors. J. Neurosurg..

[37] Peace D.J., Chen W., Nelson H., Cheever M. (1991). T cell recognition of transforming proteins encoded by mutated ras proto-oncogenes. J. Immunol..

[38] Trolle T., McMurtrey C.P., Sidney J., Bardet W., Osborn S.C., Kaever T., Sette A., Hildebrand W.H., Nielsen M., Peters B. (2016). The length distribution of class I–restricted T cell epitopes is determined by both peptide supply and MHC allele–specific binding preference. J. Immunol..

[39] Mani J., Wang L., Huckelhoven A.G., Schmitt A., Gedvilaite A., Jin N., Kleist C., Ho A.D., Schmitt M. (2017). Definition and characterization of novel HLA-*A02-restricted CD8^+^ T cell epitopes derived from JCV polyomavirus with clinical relevance. Oncotarget.

[40] Lissina A., Briceno O., Afonso G., Larsen M., Gostick E., Price D.A., Mallone R., Appay V. (2016). Priming of Qualitatively Superior Human Effector CD8(+) T Cells Using TLR8 Ligand Combined with FLT3 Ligand. J. Immunol..

[41] Neuber B., Herth I., Tolliver C., Schoenland S., Hegenbart U., Hose D., Witzens-Harig M., Ho A.D., Goldschmidt H., Klein B. (2011). Lenalidomide enhances antigen-specific activity and decreases CD45RA expression of T cells from patients with multiple myeloma. J. Immunol..

